# The Effects of an Integrated Community Case Management Strategy on the Appropriate Treatment of Children and Child Mortality in Kono District, Sierra Leone: A Program Evaluation

**DOI:** 10.4269/ajtmh.17-0040

**Published:** 2017-06-12

**Authors:** Ruwan Ratnayake, Jeffrey Ratto, Colleen Hardy, Curtis Blanton, Laura Miller, Mary Choi, John Kpaleyea, Pheabean Momoh, Yolanda Barbera

**Affiliations:** 1 Health Unit, International Rescue Committee, New York, New York;; 2 Emergency Response and Recovery Branch, Division of Global Health Protection, Center for Global Health, Centers for Disease Control and Prevention, Atlanta, Georgia;; 3 International Rescue Committee, Freetown, Sierra Leone

## Abstract

Integrated community case management (iCCM) aims to reduce child mortality in areas with poor access to health care. iCCM was implemented in 2009 in Kono district, Sierra Leone, a postconflict area with high under-five mortality rates (U5MRs). We evaluated iCCM’s impact and effects on child health using cluster surveys in 2010 (midterm) and 2013 (endline) to compare indicators on child mortality, coverage of appropriate treatment, timely access to care, quality of care, and recognition of community health workers (CHWs). The sample size was powered to detect a 28% decline in U5MR. Clusters were selected proportional to population size. All households were sampled to measure mortality and systematic random sampling was used to measure coverage in a subset of households. We used program data to evaluate utilization and access; 5,257 (2010) and 3,649 (2013) households were surveyed. U5MR did not change significantly (4.54 [95% confidence interval [CI]: 3.47–5.60] to 3.95 [95% CI: 3.06–4.83] deaths per 1,000 per month (*P* = 0.4)) though a relative change smaller than 28% could not be detected. CHWs were the first source of care for 52% (2010) and 50.9% (2013) of children. Coverage of appropriate treatment of fever by CHWs or peripheral health units increased from 45.5% [95% CI: 39.2–52.0] to 58.2% [95% CI: 50.5–65.5] (*P* = 0.01); changes for diarrhea and pneumonia were not significant. The continued reliance on the CHW as the first source of care and improved coverage for the appropriate treatment of fever support iCCM’s role in Kono district.

## INTRODUCTION

At 120.4 deaths per 1,000 live births in 2015, Sierra Leone’s under-five mortality rate (U5MR) ranks under only Angola, Chad, Somalia, and Central African Republic.^1^ Challenges in postconflict development have resulted in the failure to attain Millennium Development Goal 4 of reduced child mortality by 2015.^2^ By the end of its 10-year civil war in 2002, 50,000 persons had died and half of its population was displaced. At the war’s peak in 1996, only 16% of health centers were operational and were largely concentrated in the capital, Freetown.^3^ In 2001, the crude mortality rate (CMR) in an accessible area of the conflict-affected eastern district of Kenema was 1.2 deaths per 10,000 persons per day (95% confidence interval [CI]: 1.05–1.38), exceeding the threshold for a humanitarian crisis of one death per 10,000 persons per day.^4,5^ Most deaths were attributable to febrile illness and were therefore preventable; this indicated a collapse of the health system.

Following the war, the national health policy aimed to provide equitable health-care access through the revitalization and decentralization of front-line health facilities (called peripheral health units, or PHUs). In 2010, the Free Health Care Initiative was initiated to provide care at no cost to children under-five, pregnant women, and lactating mothers using the network of 1,200 PHUs.^6,7^ However, limited human resources remained a barrier. Approximately, 880 nurses and 825 maternal and child health aides were used at the community level, equating to 47% of the total number of community-level workers needed to meet the workload.^8^ The lack of health-care workers able to treat illnesses among children under-five continued to contribute to the high mortality.

In 2009, after providing humanitarian relief during the war, the International Rescue Committee (IRC) supported the Ministry of Health and Sanitation to develop health programs, including an integrated community case management (iCCM) program in the eastern Kono district. The main goal was to increase access to care for children under-five. The United Nations Children’s Fund and World Health Organization recommend iCCM as a strategy to promote increased and equitable access to treatment through village-based community health workers (CHWs) who treat fever, diarrhea, and suspected pneumonia among children aged 2–59 months.^9^ If iCCM can target communities with low access to care and deliver high-quality care to a high proportion of children, then in principle, rapid reductions in child mortality should follow.^10^ Although community case management for each condition has shown efficacy in reducing mortality, few evaluations of iCCM at scale have been conducted and none have demonstrated effectiveness in child survival.^10–15^ Moreover, little is known about the implementation and effects of iCCM in strengthening health systems in postconflict states, which are characterized by the degradation of government services and the workforce coupled with a distrust of services by communities.^16^ In 2010, the U5MR in Kono, as measured by a Multiple Indicator Cluster Survey, was slightly higher than the national rate, 202 versus 217 deaths per 1,000 live births, respectively.^17^ Health outcomes and health-care access were poor in 2009. The prevalence of acute malnutrition among children under-five was 8.7% and classified as medium severity of malnutrition prevalence.^17,18^ Vaccination coverage for the third diphtheria–pertussis–tetanus dose among children 12–23 months of age was low (53%), indicating poor access to PHUs and/or a low demand for services.^17^

We measured trends in process and impact indicators to investigate the effectiveness of iCCM in reducing under-five mortality and morbidity, after its introduction in September 2009. The primary objective was to measure the change in age-specific U5MRs at the midterm (in October–November, 2010) and the endline (in January–February, 2013) periods of an iCCM program which ran from September 2009 to 2013. The secondary objectives were to measure changes in care-seeking behavior; timely access to treatment; and appropriate treatment of fever, diarrhea and, suspected pneumonia among the target group of ill children aged 2–59 months. In addition, access, quality of care, level of community acceptability, and utilization over time were measured to document the program implementation.

## MATERIALS AND METHODS

### Program design, study setting, and participants.

Kono, a rural district bordering Guinea, has gold and diamond resources that made it a strategic area during the war. IRC implemented iCCM in Kono in September 2009; the program became aligned with the 2012 national policy.^19^ A high density model was used with a ratio of one CHW to 300–400 population (or to 35–50 children under-five) with CHWs based in each village providing care at all times of day. Communities selected their own CHWs to be as accessible as possible. IRC trained CHWs and supplied them with medications (artemisinin-based combination therapies [ACTs] for fever, cotrimoxazole for suspected pneumonia, and oral rehydration solution [ORS] and zinc for diarrhea), as per the national policy.^19^ CHWs treated the three diseases among children 2–59 months but did not cover other aspects of community health such as reproductive health and nutrition. Peer supervisors, former CHWs who are literate and performed well as CHWs, supervised eight to 16 CHWs. On a monthly basis, they conducted a home visit and used a checklist to assess CHW performance regarding their ability to assess a child’s respiratory rate, recall danger signs, and conduct a patient visit. Peer supervisors used the visit to review and collect data from the patient and drug registers, and to meet with community members to hear their perspectives on the CHW’s performance. They were formally supervised by the PHU in-charge and received additional supervision from an IRC health officer. Peer supervisors replenished medicines twice a month at the monthly meetings at the PHU, based on the previous month’s consumption by the CHW, and during supervision visits. They carried a small quantity of medicines in their backpack, so that they were able to replenish medicines whenever the CHW had a stock-out. There were 68 PHU catchment areas with 51,000 children under-five, supported by 831 CHWs in 14 chiefdoms in the study area. The study participants were children under-five years and persons five years and older.

### Study design.

**Figure 1.**
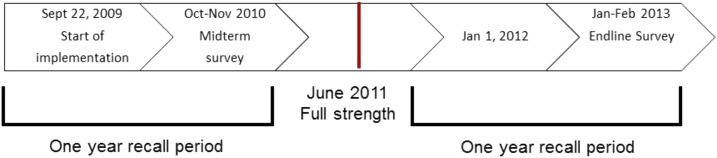
Timeline of the program implementation and evaluation, 2009–2013.

Given that this was a real time evaluation of an existing child health program, decisions on the study design were limited by logistical and ethical issues. At the project design stage, the IRC did not plan for a baseline survey and relied on the baseline mortality estimates from a survey completed in 2007 for a smaller child survival program.^20^ After implementation had begun, a midterm survey was planned in which the evaluation team wanted to account for lack of both a baseline and a control group. A traditional control group was not considered; based on the positive evidence supporting iCCM, it was expected that the intervention would be beneficial rather than harmful and withholding the intervention would be unethical.^21–23^ Furthermore, other districts were exposed to iCCM or were not similar enough at baseline to serve as a control. Therefore, a semirandomized stepped wedge design was used at midterm (1 year postimplementation) to derive baseline (preimplementation) and midterm (postimplementation) estimates of mortality. Based on the appearance of a stable trend in treatment rates, full strength implementation was achieved by June 2011. For the baseline mortality rates, the person-days started 1 year before the date of survey administration (September 22, 2009) up to the day prior to the start of the intervention date in the individual’s village. For the midterm (postimplementation) mortality rates, the person-days started at the beginning of the intervention date in an individual’s village and ended on the day of survey administration (between October 11 and November 12, 2010). However, in this paper only the midterm results mortality rates are reported and compared with the endline mortality rates. See Figure 1 for a timeline of implementation and surveys. The lack of full randomization was necessitated, as there was an ethical imperative to provide care first to the worst-off areas. Geographical areas were randomly selected from low and high health-care access groups, as the iCCM program was first implemented in PHU catchment areas with little to no access to health-care (i.e., no PHU, hospital, clinic, or pharmacy present, the distance to a PHU, hospital, clinic, or pharmacy was greater than one hour walking distance, no motorbikes or vehicles in the village, if the village was not a local trading center, and if the majority of the villagers were farmers), followed by those areas with greater access to health care.

We assessed whether the expected changes occurred after the implementation of iCCM. Multistage cluster household surveys were administered during the midterm period in 2010 and at endline period in 2013 (after an average of 8 and 34 months of implementation, respectively). The sampling frame was constructed using the number of households per village based on a previous exhaustive survey in Kono for onchocerciasis mass drug administration and further updated with the IRC’s programmatic data. The total number of households per village was used as a measure of size. Villages (clusters) were the primary sampling unit with some larger villages being divided into more than one sampling unit. In the midterm, for first stage selection, the sample was stratified into 24 PHU catchment areas. Four clusters were chosen from each PHU stratum proportional to the number of households, resulting in 96 clusters (midterm) and 90 clusters (endline). As a recall period for mortality of 12 months was used, at midterm, villages with more than 6 months without implementation were oversampled to ensure sufficient exposure time for preimplementation, and small villages were oversampled to ensure that they would not be excluded. At endline, the approach was modified; villages were classified as small (≤ 25 households), medium (26 to 50 households), or large (> 50 households), and villages with fewer than five households were excluded. Clusters were chosen from each stratum to reflect the distribution of village sizes in the sample. Medium and large villages were oversampled to decrease field visits. A household was defined as a group of people sleeping in the same structure or group of structures during the previous night and eating from the same cooking pot. In both midterm and endline surveys, second stage selection of households used one of two methods depending on the cluster size: 1) exhaustive sampling for clusters with a number of households which was equal or smaller than the expected sample size or 2) segmentation, if larger than expected. Specifically for the module on child morbidity, systematic random sampling was applied in addition to the second stage selection of households to select a subset of 13 households with children under-five from the larger sample in each cluster. Data were collected on all children under-five in the selected households. If there was no child under-five living in that household, the next household in the sampling plan was selected. Respondents were defined as mothers or caregivers of children under-five.

### Study size.

The sample size for the midterm survey was 6,067 households and was calculated to estimate the change in under-five mortality from preintervention to the midterm. The sample size was based on an estimated 30% reduction from preintervention to midterm of U5MR from a preintervention U5MR of six deaths per 1,000 children under-five per month,^20^ alpha of 0.05, power of 80%, mortality recall period of 12 months, design effect of 1.5, and a response rate of 90%. The proportion of children under-five was estimated at 17% and the average household size was estimated at six, with one child under-five per household.^17^ Of the morbidity indicators, the largest sample size required was 1,248 children or 13 children per village based on 95% CIs (±10%) and a design effect of 2. This was used as the sample size for the remaining indicators. The sample size for the endline survey was 5,125 households and was calculated to statistically detect an expected 28% decline in U5MR between the midterm and endline surveys (updated to 5,162 households as the selected clusters contained this number of households).

### Training and data collection.

Enumerators spoke a combination of multiple languages (Krio, Kono, Temne, Mende, and Fula). Each team that administered the questionnaire at a household consisted of an enumerator and a notetaker. Supervisors oversaw two to four teams during data collection in a single cluster. A 1-week training covered questionnaire administration, role-playing, and field testing. Supervisors were responsible for adherence to second stage sampling methods and assuring questionnaire completeness at the cluster. If a respondent was absent, the household was revisited twice on the same day or the following day. At the village level, if more than 15% of the households were absent, the team returned at a later date. Refusals or absences were not replaced. Investigators reviewed all questionnaires twice. If responses were found to be incomplete, the households were revisited.

### Quantitative variables, data sources, measurements, and bias.

Before designing the midterm questionnaire, focus group discussions with community members were held to better understand the definition of a household and polygamous families, orphans, multigenerational households; Krio terminology and culturally relevant ways of obtaining information on neonatal and child mortality, and signs, symptoms, and severity of the three illnesses; understanding formal and traditional sources of care, and perceptions of, and terms used for CHWs. The morbidity questionnaire was developed based on standard questions used in Demographic and Health Surveys and further modified based on pilot testing.^24^ An appropriate provider was defined as the PHU or CHW, and an appropriate treatment was defined as receiving ACT for fever, ORS and zinc for diarrhea, and amoxicillin or cotrimoxole for suspected pneumonia. The questionnaire was translated into Krio and back-translated into English and administered in Krio to an adult caregiver. For other languages, the interviewer translated the questionnaire from Krio using a list of specific standardized words that was reviewed during the training ensuring that enumerators used the same terminology. An events and agricultural calendar was used to specify salient events that could help to recall time points relevant to determine age and place deaths and events in time. The primary outcomes are described in Table 1.

**Table 1:** Primary outcomes measured by household survey

Factor	Indicator
Care-seeking behavior	Care seeking behavior from an appropriate provider by condition: proportion of ill children 2–59 months who sought care from an appropriate provider (CHW or PHU).
Coverage of appropriate treatment	Appropriate treatment from an appropriate provider (and CHW only) by condition: proportion of ill children 2–59 months who sought care from an appropriate provider (CHW or PHU/CHW only), who received ACT for fever, ORS, and zinc for diarrhea or amoxicillin or cotrimoxole for pneumonia.
Access to treatment	Timely treatment within 24 hours of onset of symptoms.
Impact among target group (and comparison groups)	Mortality rate among children 2–59 months: Number of children 2–59 months who died per 1,000 children 2–59 months per month.
	Mortality rate among children under-five: Number of children under-five who died per 1,000 children under-five per month.
	Mortality rate among persons 5 years and older: Number of persons 5 years and older who died per 1,000 persons per month.
Community acceptability	Recognition of the CHW: proportion of households whose respondent could correctly name the CHW.
Quality of care	Correct case management: proportion of illnesses among children 2–59 months where the CHW administered an appropriate treatment or referred child to a PHU.

ACT = artemisinin-based combination therapy; CHW = community health worker; ORS = oral rehydration solution; PHU = peripheral health unit.

For the mortality questionnaire, a hybrid current and past household census was undertaken in which all members present at the start and end of the recall period as well as births and deaths were recorded to account for in- and out- migration.^25^ This used a recall period of 1 year to estimate age-specific mortality rates for the target group (2–59 months of age). Two groups were analyzed (under-five including those less than 2 months of age; 5 years and over) to understand background mortality estimates among the population that iCCM was not intervening on. Given the high levels of in- and out- migration in mining communities and other areas and the need for precision around the exposure period to account for the staggered program implementation, a hybrid household census provided the most accurate accounting of exposure time in the denominator. Age-specific mortality rates that measure incidence of death over a 12-month recall period were used instead of the previous birth history approach that is typically used in national surveys. A previous birth history tracks all births to a mother in her lifetime to produce the lifetime probability of dying before the age of five, regardless of her place of residence inside or outside the program catchment area.^5,25^ Age-specific mortality rates were used as the program was applied to a specific catchment area, population, time. Therefore, exposure to the program would be most likely if a defined limited recall period was used. The use of a limited recall period also improves the accuracy of recall of the vital events and their dates, whereas a full birth history has been shown to substantially underestimate under-five mortality.^5^

After conducting the midterm survey, potential biases were identified and addressed (see Table 2). The midterm results indicated that communities in peri-urban settings were reliant on health-care facilities and private clinics rather than CHWs; therefore, the IRC stopped supporting iCCM in two peri-urban areas (these clusters were removed from the midterm analysis and the endline sampling frame). During the midterm, respondents identified medicines received by pointing to pictures of the iCCM drugs, whereas actual samples of blister packs of drugs used by iCCM and blister packs and bottles used by the PHUs were shown to respondents at endline. The case definition for suspected pneumonia at midterm required caregivers to spontaneously report fast or difficult breathing when requested to describe the child’s symptoms. This produced very few suspected pneumonia cases. At endline, the requirement for the caregiver to first report fast or difficult breathing was modified to increase the sensitivity of the case definition. Therefore, if a cough or cold were reported, respondents were prompted about other symptoms including fast breathing. Since the method of measuring suspected pneumonia changed between rounds, the indicators are not directly comparable and only the endline results for treatment are reported in this paper.

**Table 2:** Key differences between midterm and endline surveys

	Midterm	Endline
Objective	Measure mortality change between preimplementation to postimplementation phase	Measure mortality change between midterm and endline
Survey design	Multistage cluster survey with a semirandomized, stepped wedge component	Multistage cluster survey
Sample size	6,067 households, 96 clusters	5,125 households, 90 clusters
Stratification	PHU	Small, medium, large villages
Sampling	Sampled urban areas (excluded in the analysis)	No urban areas sampled
Data collection	Respondents referred to treatments using a picture card	Respondents referred to treatments using samples of actual treatments (bottles, blister packs)
	Pneumonia case definition required that respondents reported fast or difficult breathing	Pneumonia case definition modified so that if a cough/fast or difficult breathing/cold were reported, respondents were prompted about the other symptoms

### Statistical methods.

The data were entered into a Microsoft Access database using double entry and error checks. SAS 9.3 (SAS Institute Inc., Cary, NC) and SUDAAN (Research Triangle Institute, Research Triangle Park, NC) were used to compute complex survey procedures to account for the survey sample weights, stratification, and clustering. CIs (95%) were computed using a *t* value with an alpha of 0.05 and a degree of freedom equal to the number of clusters minus the number of strata. Sampling weights were calculated to account for unequal probabilities of selection and nonresponse. Weighted proportions were calculated for the morbidity-related variables and to account for differences between proportions of children under-five between rounds. Multiple symptoms per child were reported and analyzed. To test for significant differences between morbidity-related variables, χ^2^ tests were used. Mortality rates were calculated for the entire recall period. The number of person-days started at the beginning of the recall period, or when the individual joined the household after that date, and were converted into months. Person-days ended the day of the survey or exit from the household by death or out-migration. To test significance between mortality rates, a *t* test of the linear contrast was used.

### Program implementation.

Routine program data and additional survey indicators were analyzed to document program implementation.^26^ To address access to care during the 4-year project period, the yearly under-five population to CHW ratio (equivalent to, [under-five population/number of CHWs]) was sourced from routine program data and calculated and compared with target of 35–50 children under-five. To assess utilization over time, the treatment rate, or mean treatments per under-five population per month by condition and by source, were sourced from routine program data and analyzed for the period covered by the survey, October 2009 to February 2013 (equivalent to, [number of treatments per month/under-five population] × 1,000 children under-five). Stata 14.0 (StataCorp LP, College Station, TX) was used to calculate, by condition and source, the differences in means using an unpaired *t* test and to assess the trend with linear regression. To assess community recognition of the CHW, survey data on the proportion of households that could correctly report the name of the CHW providing iCCM was assessed (Table 1). For quality of care, survey data on the proportion of fever, diarrhea, and suspected pneumonia cases who were appropriately managed among children who sought care from a CHW were analyzed (Table 1). Appropriate management included appropriate treatment of each condition, or referral when the child was less than 2 months, treatment was unavailable or conditions were associated with danger signs.

### Ethical review.

The evaluation received ethical approval from the Office of the Sierra Leone Ethics and Scientific Review Committee. The Centers for Disease Control and Prevention’s Institutional Review Board determined the evaluation to be a nonresearch activity. Oral informed consent was obtained from each respondent.

## RESULTS

### Participants.

Data collection were undertaken between October 11 and November 12, 2010, and January 26 and February 20, 2013. At midterm, 96 of 96 clusters containing 5,257 households were surveyed. During the analysis phase, two clusters containing 89 households were excluded because they were found to be outside of the project area. At endline, 89 of 90 clusters containing 3,649 households were surveyed. In error, one cluster was listed twice in the sampling frame in two different, bordering chiefdoms. Two clusters were found to be outside the intervention area and were replaced randomly with two clusters from the same size strata. In both surveys, the primary reason for missing households was sampling frame errors where village population sizes were lower than expected. This resulted in a reduction to 86.6% and 70.7% of the target sample size in the midterm and endline evaluations, respectively. During both surveys, 95% of the visited households responded. The demographic characteristics of the two survey samples were similar (Table 3).

**Table 3:** Demographic characteristics, 2010 and 2013

	Midterm	Endline
Demographics	*N*	*N*
Number of households	5,257	3,649
Population recorded	32,808	28,914
Children under-five years (% of total population)	5,257 (16%)	3,869 (16.4%)
Mean household size	6.7	6.6
Mean number of children under-five per household	1.7	1.6

### Mortality.

Including neonates not targeted by iCCM, the U5MR did not change significantly from the midterm to the endline survey, from 4.54 (95% CI: 3.47–5.60) to 3.95 (95% CI: 3.47–5.60) deaths per 1,000 children under-five per month, respectively (*P* = 0.4). The mortality rates did not change significantly for the 2–59 months age group targeted by iCCM (decreasing from 3.64 (95% CI: 2.79–4.49) to 2.88 (95% CI: 2.28–3.47) deaths per 1,000 per month in 2010 and 2013, respectively) (*P* = 0.15; Table 4). Mortality rates among persons 5 years and older not targeted by iCCM increased from 0.68 (95% CI: 0.54–0.81) deaths to 0.93 (95% CI: 0.75–1.11) deaths per 1,000 persons five and over per month (*P* = 0.03, 37% increase [95% CI: 2.9–70.6]).

**Table 4:** Under-five mortality rates, 2–59 month mortality rates and 5 years and over mortality rates, 2010 and 2013

Mortality	Midterm				Endline				Change	
	Mortality rate (95% CI)^*^	DEFF	Deaths	*N* (persons)	Mortality rate (95% CI)*	DEFF	Deaths	*N* (persons)	% Change	*P* value
0–59 months^*^	4.54 (3.47–5.60)	2.27	146	3,466	3.95 (3.06–4.83)	1.99	184	3,869	−13% (−43.2 to 17.2)	0.4
2–59 months^*^	3.64 (2.79–4.49)	1.86	116	3,313	2.88 (2.28–3.47)	1.27	143	3,665	−21% (−49.5 to 7.7)	0.15
≥ 5 years^*^	0.68 (0.54–0.81)	1.86	131	17,375	0.93 (0.75–1.11]	2.09	213	20,045	+37% (2.9 to 70.6)	0.03

CI = confidence interval; DEFF = design effect.

* Rate is per 1,000 persons per month.

### Coverage, care-seeking behavior, timely treatment, and appropriate treatment.

All coverage indicators refer to children 2–59 months of age. Among all ill children, the CHW as the first source of care remained unchanged: 52% (95% CI: 45.3–58.6) in 2010 and 50.9% (95% CI: 41.2–60.4) in 2013 (*P* = 0.85). The PHU as the first source of care decreased from 30% (95% CI: 24.8–35.9) to 21.7% (95% CI: 17.4–26.8) (*P* = 0.02). Taken together, the first source as an appropriate provider (CHW or PHU) decreased from 82% (95% CI: 75.8–86.9) to 72.6% (95% CI: 64.0–79.7) (*P* = 0.03). The proportion of ill children for whom no treatment was sought remained similar at 3.9% (95% CI: 2.1–7.3) in 2010 and 4.7% (95% CI: 3.0–7.4) in 2013 (*P* = 0.63). At endline, a treatment (either appropriate or not appropriate) was received within 24 hours for approximately 83% (95% CI: 77.8–87.1) of fever episodes, 82.9% (95% CI: 67.4–91.9) of diarrhea episodes, and 78.8% (95% CI: 66.0–87.7) of suspected pneumonia episodes.

**Figure 2.**
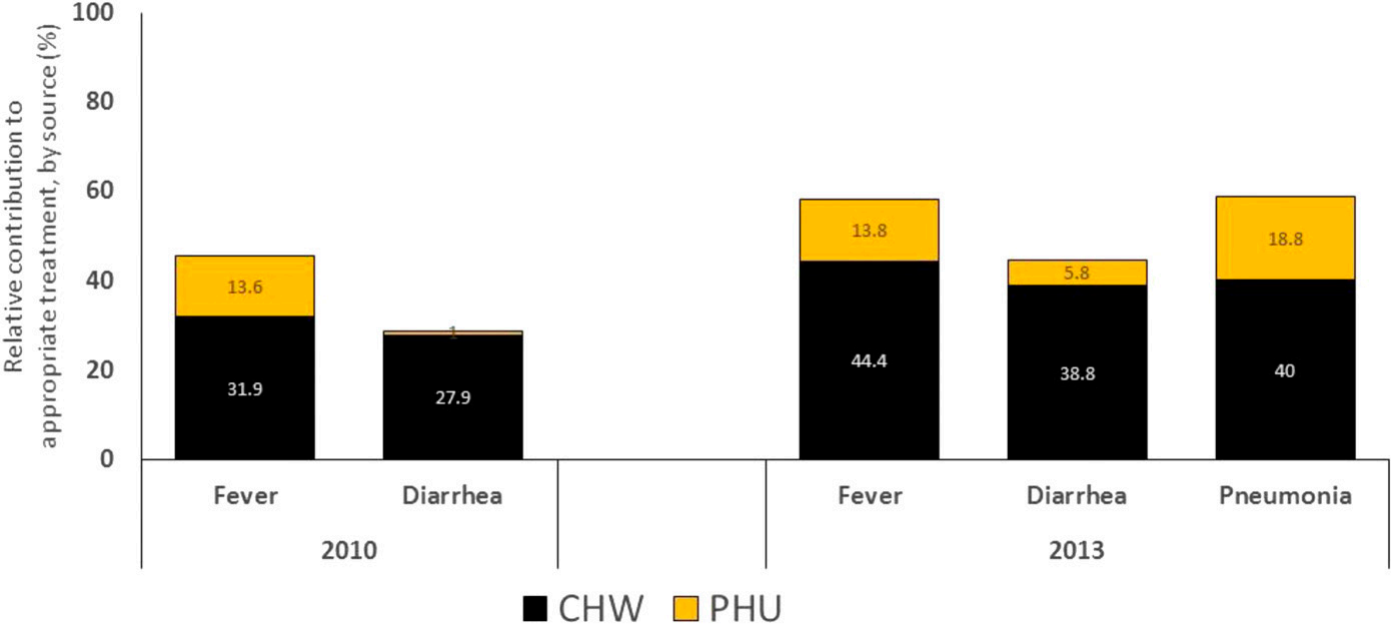
Relative contribution of CHWs and peripheral health units (PHUs) to delivery of appropriate treatment, 2010 and 2013.

Table 5 presents the care-seeking and coverage indicators by condition. There was a decrease in the proportion of children with fever who sought treatment from an appropriate provider, from 83.2% (95% CI: 76.7–88.2) in 2010 to 72.4% (95% CI: 62.6–80.5) in 2013 (*P* = 0.02). Appropriate treatment of fever from an appropriate provider and CHW increased from 45.5% (95% CI: 39.2–52) to 58.2% (95% CI: 50.5–65.5) (*P* = 0.01) and 31.9% (95% CI: 26.7–37.6) to 44.4% (95% CI: 36.2–52.8) (*P* = 0.01), respectively. Care-seeking for diarrhea from an appropriate provider remained consistently high across rounds (87.3% [95% CI: 70.3–95.3] and 83.0% (95% CI: 68.6–91.6]). Appropriate treatment from an appropriate provider for diarrhea did not increase significantly and was 28.9% (95% CI: 14.2–50) at baseline and 44.6% (95% CI: 33.9–55.8) at endline, nor from the CHW, at 27.9% (95% CI: 13.2–49.5) at baseline and 38.8% (95% CI: 27.2–51.8) at endline. At endline, care-seeking from appropriate provider for suspected pneumonia was 86.7% (95% CI: 66.8–95.5) and appropriate treatment from an appropriate provider and the CHW was 58.8% (95% CI: 41.1–74.5) and 40.0% (95% CI: 25.4–56.6), respectively. We considered the sample sizes for pneumonia at midterm to be too small to test for significant differences. The relative contributions of CHWs and PHUs to appropriate treatment are shown in Figure 2. At endline, CHWs contributed 76.2%, 87.0%, and 68.0% of all appropriate treatments delivered for fever, diarrhea, and suspected pneumonia, respectively.

**Table 5:** Number and proportion of ill children receiving treatment within 24 hours of symptom onset and number and proportion of ill children seeking and receiving treatment from an appropriate provider (CHW or government clinic), 2010 and 2013

Total children	Midterm		Endline		*P* value
	*N* = 1,226		*N* = 904		
	*N*	% (95% CI)	*N*	% (95% CI)	
Fever	415	81.6 (76.6–85.7)	439	75.5 [70.8–79.7]	0.06
Received any treatment ≤ 24 hours of symptom onset	334	79.4 (73.4–84.4)	367	83 (77.8–87.1)	0.34
Sought treatment from appropriate provider	350	83.2 (76.7–88.2)	344	72.4 (62.6–80.5)	0.02
CHW/PHU					
Appropriate treatment*	200	45.5 (39.2–52)	273	58.2 (50.5–65.5)	0.01
CHW					
Appropriate treatment*	148	31.9 (26.7–37.6)	219	44.4 (36.2–52.8)	0.01
Diarrhea	48	8.7 (6.5–11.7)	90	15 (11.9–18.8)	0.003
Received any treatment ≤ 24 hours of symptom onset	42	91.7 (80.1–96.8)	79	82.9 (67.4–91.9)	0.18
Sought treatment from appropriate provider	42	87.3 (70.3–95.3)	78	83 (68.6–91.6)	0.6
CHW/PHU					
Appropriate treatment*	15	28.9 (14.2–50)	47	44.6 (33.9–55.8)	0.18
CHW					
Appropriate treatment*	14	27.9 (13.2–49.5)	44	38.8 (27.2–51.8)	0.37
Pneumonia†	6	‡	76	13.1 (9.9–17.1)	†
Received any treatment ≤ 24 hours of symptom onset	3	‡	60	78.8 (66–87.7)	†
Sought treatment from appropriate provider	5	‡	68	86.7 (66.8–95.5)	†
CHW/PHU					
Appropriate treatment*	0	0	49	58.8 (41.1–74.5)	†
CHW					
Appropriate treatment*	0	0	36	40 (25.4–56.6)	†

ACT = artemisinin-based combination therapy; CHW = community health worker; PHU = peripheral health unit.

* Appropriate treatment indicates ACT (fever), ORS and zinc (diarrhea), amoxicillin or cotrimoxazole (pneumonia).

† We consider the sample sizes too small to ascertain a significant difference.

‡ Numbers too small to calculate.

### Program implementation.

**Figure 3.**
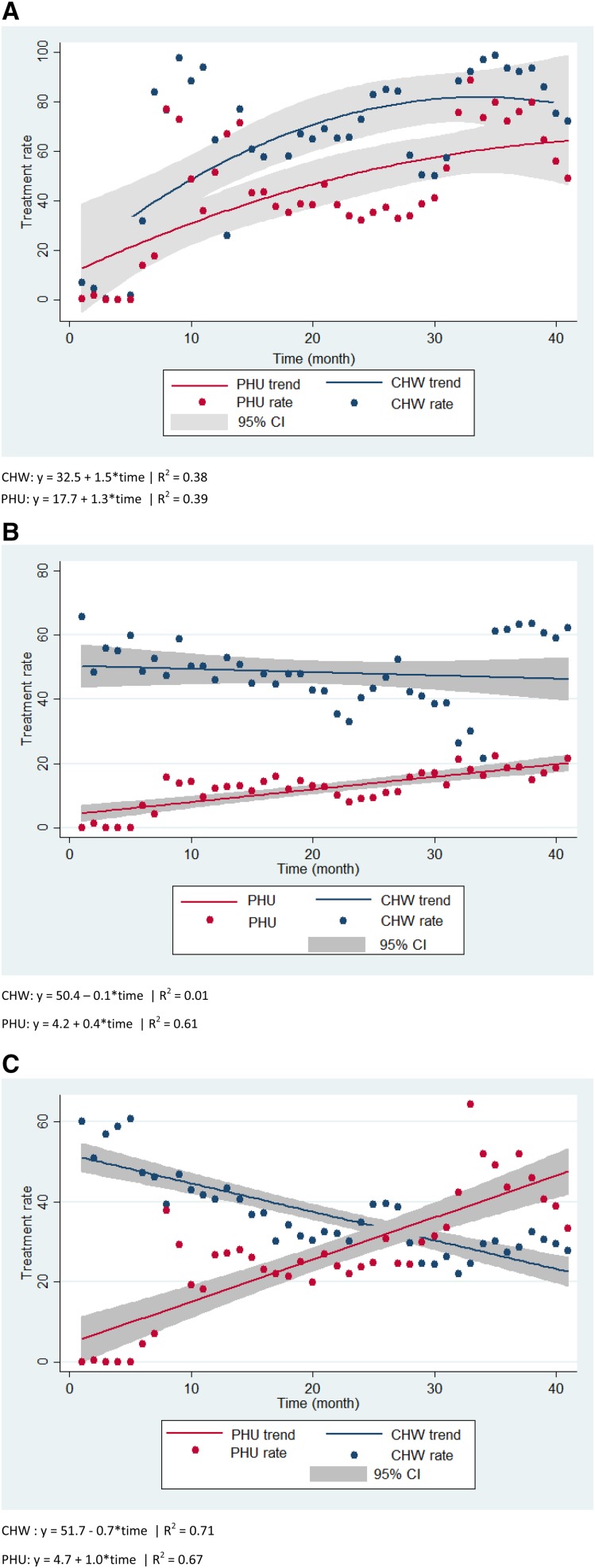
(**A**–**C**). Treatment rate and linear trend for fever, diarrhea, and pneumonia, per 1,000 children under-five and by month, administered by CHWs and PHUs, October 2010–February 2013. (**A**) Fever. (**B**) Diarrhea. (**C**) Pneumonia. CHW = community health worker; PHU = peripheral health unit.

In terms of access, the average population under-five to CHW ratio increased from 38 (2009) to 67 (2013), higher than the anticipated ratio of 35–50. For utilization, there are considerable variability in the data. CHWs had mean treatment rates that were 10–36 treatments per under-five population per month higher than of PHUs across all conditions from October 2009 to February 2013 (all conditions, *P* < 0.001; Table 6). Figure 3A–C show a more stable trend in monthly treatment rates for fever toward the midpoint of the program, and some consistency in diarrhea treatments among CHWs that are generally higher than that of PHUs. The trend for monthly suspected pneumonia treatment rates among CHWs descends in contrast to an ascending trend among PHUs. For recognition of the CHW, the survey results showed there was an increase in recognition of the CHW from 57.5% to 82.4% (*P* < 0.005) households who could name the CHW without prompting. For quality of care, the proportion of ill children who were appropriately managed by the CHWs increased from 62.4% (95% CI: 55.4–68.9) to 83.6 (95% CI: 79.1–87.2) (*P* < 0.001).

**Table 6:** Mean treatment rates for CHWs and health facilities, October 2010 to February 2013

	PHU	CHW	Difference	*P* value*
Fever	44.7	64.0	19.3	0.0017
Diarrhea	12.4	48.3	35.9	0.0000
Pneumonia	26.7	36.8	10.1	0.0007

CHW = community health worker; PHU = peripheral health unit.

* Unpaired, two-tailed *t* test for two samples.

## DISCUSSION

This program evaluation examined the effects of a high-density iCCM program on care-seeking behavior, timely treatment, appropriate treatment, and mortality among children under-five in Kono district, a rural, postconflict area with low access to care. Utilization of the CHW as the first source of care remained at 50% in both rounds. However, the already high proportion of households that could correctly name the CHW without prompting increased from 58% to 82%. At endline, 73% of ill children first sought care from either the CHW or PHU, which decreased from 82% at midterm. The decrease was likely driven by a decrease in seeking care from the PHU from midterm to endline. At endline, the receipt of timely treatment was high across conditions (80%), however, the ratio of CHWs to children (1:67) fell below the target (1:50) but remained at the low end of the range calculated in a study of 15 iCCM programs (mean 328; median 94; range 35–2,007).^27^ Taken together, this indicates that the program provided timely and widely distributed access to treatment. In terms of quality of care, 84% of all conditions were appropriately managed at endline compared with 62% at midterm, signaling that the capacity to manage cases improved. The survey-based coverage estimates are backed by mean monthly treatment rates by CHWs that were 10–36 treatments per under-five population per month higher than that of PHUs.

At 72% (fever), 83% (diarrhea), and 87% (pneumonia) at endline, care-seeking from an appropriate provider is considerably higher than that demonstrated during another endline survey evaluating care-seeking from appropriate providers for all conditions in Kambia and Pujehun districts of Sierra Leone (57.1%).^28^ It is notable that this evaluation identified a high proportion of children with fever across both rounds (34% and 49%) accompanied by a significant 13% point increase from 45% to 58% in the coverage of appropriate treatment of fever from an appropriate provider. CHWs contributed to 44% of the 58% of the coverage of appropriate treatment of fever at endline, the larger proportion versus PHUs. The coverage of appropriate treatment of suspected pneumonia was nearly 60% at endline, with CHWs contributing 40% of the 60%. In contrast, the coverage of appropriate treatment at endline for diarrhea was low at 45%, though CHWs provided 39% of the 45%. The lower diarrhea treatment estimates are similar to lower than anticipated diarrhea treatments from diverse settings including Côte d’Ivoire, Rwanda, and South Sudan.^29^ Low numbers of diarrhea cases were found in the midterm and endline surveys, which may indicate poor reporting of diarrhea from the community. This possibly results from a belief that diarrhea is not life-threatening and the complex and incorrect understandings of causation and the prevention of diarrhea.^29,30^ Nevertheless, at endline, the measured coverage of appropriate treatment were not as high as the care-seeking estimates. This is likely due to measurement error related to the caregivers not being able to recall accurately the exact treatments used by CHWs and PHUs based on viewing sample blister packs and bottles at the time of survey administration.^31^ As well, PHUs and CHWs may not have given appropriate treatments for a given condition. However, CHWs are only stocked with appropriate treatments, making this possibility less likely. Alternative explanations are not supported by routine data; during the 2-week morbidity recall period for the endline survey, there were no missing CHWs and the percentage of CHWs reporting a full stock of drugs was 100% (Ratnayake R, unpublished data). Overall, the proportions of appropriate treatment achieved for all conditions are comparable for fever and higher for other conditions than those seen in the iCCM-treatment group in a recent evaluation of iCCM in Central Uganda (64.3%, fever; 28.8%, pneumonia; 15.9% diarrhea).^32^

Overall, the U5MR detected was lower than expected. The 13% decline in mortality rates for children under-five was not statistically significant. This result remains inconclusive as the sample size was not powered to detect a relative change smaller than 28% and the U5MR across rounds was lower than anticipated. This is a result of logistical and financial constraints common to evaluating in real time child health programs, where it is unfeasible to use the very large sample sizes needed to account for the short implementation periods in which to accumulate a rare event like deaths.^10^ However, we found that the trends in timely and equitable access, appropriate treatment, quality of care, community recognition of the CHW, and utilization over time showed the expected progress, indicating that the reduction of morbidity is likely. There were also decreases in severe acute malnutrition in Kono during the same period from 3.9% (2010) to 1.1% (2013), which are congruent with lower child mortality.^17,33^ Of note, mortality among persons 5 years and older increased significantly by 37%. Adult mortality rates have been decreasing in Sierra Leone and other African countries, whereas maternal mortality remains high in Sierra Leone.^34,35^ Even with the increase in mortality among persons 5 years and older observed in the endline survey, the CIs of the midterm CMR (0.46/10,000/day, 95% CI: 0.38–0.53) and endline CMR (0.41/10,000/day, 95% CI: 0.34–0.48) overlap with the Sphere Project’s baseline CMR for sub-Saharan Africa (0.41/10,000/day).^36^ The recall period of the endline survey coincided with a national cholera outbreak in which Kono was affected.^37,38^ However, we did not carry out a verbal autopsy to determine the causes of death which may have been helpful for developing a hypothesis about potential causes and demographic groups contributing to this increase.

It is notable that several other evaluations of scaled iCCM programs in Burkina Faso, Ethiopia, and Malawi over the same implementation period have not demonstrated meaningful increases in care-seeking and coverage of appropriate treatments.^11,12,15^ This was related to program inputs and contextual factors that affected coverage and impact.^39^ It is therefore useful to note that the findings of the midterm survey led to improvements in program implementation. First, as part of the investigation into the mortality estimates, an audit into drug availability among CHWs between September 2009 and November 2010 found that CHWs were provided a set amount of drugs that did not match demand, resulting in regular drug stock-outs in villages. Owing to central stock-outs in Kono or inefficient re-supply of CHW stocks, ACT, co-trimoxazole and ORS and zinc were available, respectively, during 45%, 68%, and 69% of the total days (Ratnayake R, unpublished data). Given that malaria contributes substantially to the burden of disease, it is likely that the stock-out levels had a substantial negative impact on child mortality during the postintervention period. As a result, the IRC tripled the drug supply to CHWs, and continually restocked CHWs based on the previous month’s consumption. Supervisors also began monitoring the risk of CHWs not having enough medicine to provide a complete treatment of 24 hours. Second, early in the project cycle, detection of CHWs’ overtreatment of simple cough in children led to corrective efforts to minimize antibiotic treatment of cough; this may help to explain the decrease in treatment by CHWs of suspected pneumonia over time. Third, CHWs were withdrawn from peri-urban areas, and the community that immediately surrounds PHUs as data showed a low utilization on CHWs in these areas. Locations of CHWs were remapped to ensure that all hard to reach areas had a CHW, and resources were focused on the hardest to reach areas.

There are several important limitations to this evaluation. The drug stock-outs likely led to higher child mortality rates in the postimplementation period, making them less comparable with the preimplementation period. Therefore, we chose to compare midterm (postimplementation) and endline mortality rates and to exclude the preimplementation baseline mortality rates. In addition, without a baseline survey of morbidity, we can only compare endline (full intervention) with midterm (medium strength intervention) coverage estimates. Therefore, it is possible that the measurement of larger effects of iCCM was missed. The sampling frame contained instances of erroneous village sizes that contributed to a 30% reduction in sample size at the endline. Since the method of measuring suspected pneumonia changed between rounds, the indicators are not directly comparable and few cases were detected at midterm. However, problems with the discriminative power and specificity of the suspected pneumonia case definition have been recognized and there is currently no solution for producing valid treatment rates.^40,41^ Surveys are subject to difficulties with accurate recall of the source of care and types of treatments.^42^ To minimize this effect, we asked caregivers to recall whether the provider carried a drug box with the IRC logo. We asked caregivers to identify treatments without prompting by using pictures (at midterm) or packages (at endline) from the array of all treatments to avoid the misspecification of treatments caused by the interviewer reading out a list of treatments. Using our approach, the two treatments administered for diarrhea (ORS and zinc) that signify appropriate treatment may have been difficult to identify jointly and therefore left out of the numerator.^43^ Differences between the two surveys (small villages oversampled in midterm and large villages oversampled in endline) may impact results. The length of time for implementation between surveys was too short to see very large changes in mortality (although there was a fairly large but nonsignificant decrease noted). Finally, the programmatic data is limited by the availability of accurate population data and data quality issues inherent to data collected routinely.^29^

Rehabilitation of health systems in the postconflict period can benefit from community-based approaches. The 2014–2016 Ebola outbreak also demonstrated the potential for CHWs to enable more resilient health systems through their inherent roles in mobilizing and engaging their own communities and in extending surveillance to the village level.^44–46^ iCCM continues to be a realistic strategy to reducing the treatment gap for children in the most rural and hard to reach areas of Kono District. Lower than anticipated diarrhea treatment rates should be addressed and efforts to increase care-seeking at both the CHW and PHU level should be undertaken. Overall, iCCM provides a counterpart to the Free Health Care Initiative as it overcomes barriers of access to health facilities and provides insurance against the national stock-out of medicines.
